# Multicomponent Click Synthesis of New 1,2,3-Triazole Derivatives of Pyrimidine Nucleobases: Promising Acidic Corrosion Inhibitors for Steel

**DOI:** 10.3390/molecules181215064

**Published:** 2013-12-06

**Authors:** Rodrigo González-Olvera, Araceli Espinoza-Vázquez, Guillermo E. Negrón-Silva, Manuel E. Palomar-Pardavé, Mario A. Romero-Romo, Rosa Santillan

**Affiliations:** 1 Departamento de Ciencias Básicas, Universidad Autónoma Metropolitana, Av. San Pablo No. 180, México D.F., C.P. 02200, Mexico; E-Mails: rogo@correo.azc.uam.mx (R.G.-O.); arasv_21@hotmail.com (A.E.-V.); 2 Departamento de Materiales, Universidad Autónoma Metropolitana, Av. San Pablo No. 180, México D.F., C.P. 02200, Mexico; E-Mails: mepp@correo.azc.uam.mx (M.E.P.-P.); mmrr@correo.azc.uam.mx (M.A.R.-R.); 3 Departamento de Química, Centro de Investigación y de Estudios Avanzados del Instituto Politécnico Nacional, Apartado Postal 14-740, 07000 México D. F., Mexico; E-Mail: rsantill@cinvestav.mx

**Keywords:** nucleobases, multicomponent reaction, 1,2,3-triazoles, acidic corrosion, steel

## Abstract

A series of new mono-1,2,3-triazole derivatives of pyrimidine nucleobases were synthesized by one-pot copper(I)-catalyzed 1,3-dipolar cycloaddition reactions between *N*-1-propargyluracil and thymine, sodium azide and several benzyl halides. The desired heterocyclic compounds were obtained in good yields and characterized by NMR, IR, and high resolution mass spectrometry. These compounds were investigated as corrosion inhibitors for steel in 1 M HCl solution, using electrochemical impedance spectroscopy (EIS) technique. The results indicate that these heterocyclic compounds are promising acidic corrosion inhibitors for steel.

## 1. Introduction

1,2,3-Triazoles are present in a number of compounds with assorted biological activities such as anticancer, antibacterial, antifungal, anti-tubercular, and anti-HIV properties [1,2]. Nowadays, the copper(I)-catalyzed azide-alkyne cycloaddition (CuAAC, also known as the copper(I)-catalyzed Huisgen-Meldal-Sharpless cycloaddition) is the most widely used method for the synthesis of 1,4-disubstituted 1,2,3-triazoles from a wide range of organic azides and terminal alkynes [3,4,5,6,7]. Moreover, this process allows for the assembly of complex molecules, thus generating new unknown structures with an added potential biological and engineering value [8,9,10]. Monopropargyl pyrimidine nucleobases (uracil and thymine) are versatile building blocks for the synthesis of biologically relevant 1,2,3-triazoles [11]. They are generally used as starting material for the synthesis of triazole nucleosides [12,13,14,15,16,17,18,19], triazole nucleotides [20,21,22,23], oxiconazole analogues [24], nucleopeptides [25], inhibitors of human topoisomerase type II [26], and nucleoamino oxyacids [27]. Further, these propargyl nucleobases are also used in the synthesis of organogels [28], and as corrosion inhibitors [29]. In the last years, the corrosion inhibition of steel in acid solutions by nitrogen-containing heterocyclic compounds has been extensively studied. In this regard, 1,2,4-triazole derivatives are considered to be effective acidic corrosion inhibitors [30,31,32,33,34]. Recently, some 1,2,3-triazole derivatives have been reported as a new class of corrosion inhibitors in acidic media [35,36,37,38,39].

To continue with our project on the synthesis of organic inhibitors for acidic corrosion of steel grade API 5L X52 [29], a series of new 1,2,3-triazole derivatives of nucleobases which incorporate the known structural features of corrosion inhibitory activity such as pyrimidine nucleobases (uracil and thymine) [40], and the 1,2,3-triazole moiety [35,36,37,38,39] were synthesized. This class of nitrogen heterocyclic compounds is of particular interest because of their promising corrosion inhibitory activity.

## 2. Results and Discussion

### 2.1. Synthesis

**Scheme 1:**
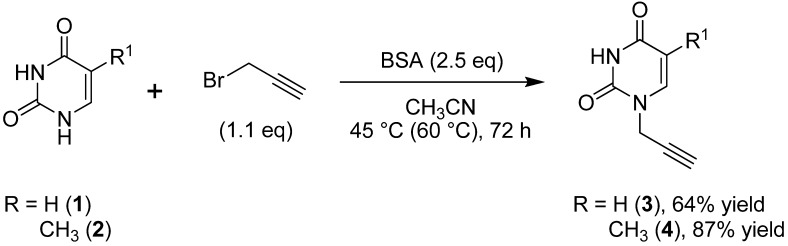
Propargylation of pyrimidine nucleobases **1**–**2**.

Propargyl nucleobases **3**–**4** are accessible after just one preparation step starting from the corresponding uracil and thymine with propargyl bromide under basic conditions (K_2_CO_3_ or DBU) [41,42,43] or employing bis(trimethylsilyl)pyrimidine nucleobase [44,45,46]. Due to the feasibility of performing selective alkylation at *N*-1, the propargylation of bis(trimethylsilyl)pyrimidine method was selected to prepare compounds **3**–**4**. Thus, uracil was treated with *N*,*O*-bis-(trimethylsilyl)acetamide (BSA) and propargyl bromide in dry acetonitrile under different conditions. Stirring the reaction mixture for 11 days at room temperature provided the desired product **3** in low yield (20%). When refluxing in dry CH_3_CN for 3 h was attempted, the TLC (CH_2_Cl_2_/MeOH, 95:5 *v*/*v*) showed a mixture of *N*-1-propargyluracil **3** and 1,3-dipropargyluracil. The optimum conditions for the propargylation reaction involved stirring at 45 °C for 72 h. Here, the 1,3-dipropargyl uracil was not observed in the reaction (TLC) and the desired product **3** was obtained in 64% yield after workup and purification by recrystallization (Scheme 1). Similarly to **3**, the desired product *N*-1-propargylthymine **4** was prepared and isolated in 87% yield (Scheme 1).

With compounds **3**–**4** in hand, we then performed a one-pot three-component 1,3-dipolar cycloaddition reaction [47,48,49,50] to generate a series of 1,4-disubstituted 1,2,3-triazole nucleobases. Based on our previously reported methodology [29,51], the reaction between monopropargyl nucleobases **3**–**4**, sodium azide, and several benzyl halides was carried out in the presence of a catalytic amount of Cu(OAc)_2_∙H_2_O in EtOH-H_2_O (2:1 *v*/*v*) at room temperature for 24 h to give the desired products **5**–**14** in good yields (Table 1).

**Table 1:** One-pot three-component click reaction. 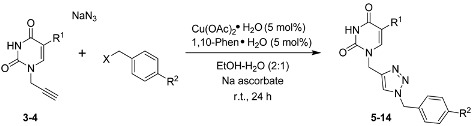

Entry	Compound	R^1^	R^2^	X	Yield ^a^ (%)
1	5	H	H	Cl	84
2	6	H	F	Cl	90
3	7	H	Cl	Cl	80
4	8	H	Br	Br	83
5	9	H	I	Br	81
6	10	CH_3_	H	Cl	81
7	11	CH_3_	F	Cl	90
8	12	CH_3_	Cl	Cl	87
9	13	CH_3_	Br	Br	83
10	14	CH_3_	I	Br	85

^a^ Isolated yields after purification.

The structures of the prepared compounds were confirmed by ^1^H- and ^13^C-NMR spectroscopic methods, and mass spectra. The ^1^H and ^13^C-NMR signals for 1,2,3-triazole nucleobases **5**–**14** were assigned with the help of standard 2D heteronuclear correlation method (Table 2 and Table 3). A singlet observed in the ^1^H-NMR spectrum at δ = 8.10–8.13 ppm confirmed the presence of the triazolyl hydrogen, supported by the signals in the ^13^C-NMR spectrum at δ = 124.1–124.3 ppm. The signals for the quaternary carbon of the triazole ring appeared at δ =143.2–143.5 ppm in the ^13^C-NMR spectrum. These chemical shift values are consistent with those reported for 1,4-disubstituted 1,2,3-triazoles [29,51,52].

The signals of the aromatic carbons in compounds **6** and **11** can be readily assigned based on their *J*_CF_ coupling constants (Table 2 and Table 3). For example, the ^13^C-NMR spectrum for compound **6** shows four doublets at 162.4, 116.1, 130.9, and 132.7 ppm with values of *J*_CF_ = 244.0 (^1^*J*), 21.4 (^2^*J*), 8.8 (^3^*J*), and 2.5 (^4^*J*) Hz, respectively.

**Table 2:** ^1^H, and ^13^C-NMR chemical shifts (ppm) for compounds **5**–**9** in DMSO-*d*_6_. 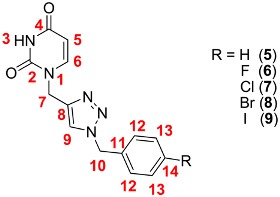

		3-H		5-H	6-H	7-H		9-H	10-H		12-H	13-H	14-H
	2-C		4-C	5-C	6-C	7-C	8-C	9-C	10-C	11-C	12C	13-C	14-C
**5**		11.29		[a]	7.71	4.89		8.10	[a]		[a]	[a]	[a]
	151.3		164.3	101.8	146.1	43.0	143.2	124.2	53.4	136.4	128.5	129.3	128.7
**6**		11.28		5.55	7.71	4.89		8.10	5.53		7.36	7.17	---
	151.3		164.2	101.8	146.1	43.0	143.3	124.1	52.5	132.7	130.9	116.1	162.4
**7**		11.28		5.55	7.71	4.89		8.11	5.54		7.30	7.40	---
	151.3		164.2	101.8	146.1	43.0	143.3	124.3	52.5	135.4	130.5	129.3	133.4
**8**		11.29		5.55	7.70	4.88		8.11	5.52		7.23	7.53	---
	151.3		164.3	101.8	146.1	43.0	143.3	124.3	52.6	135.8	130.8	132.2	122.0
**9**		11.28		5.50	7.70	4.88		8.10	5.50		7.08	7.70	---
	151.3		164.3	101.8	146.1	43.0	143.2	124.3	52.7	136.2	130.8	138.1	95.1

[a] See Experimental section.

### 2.2. Corrosion Inhibition Efficiencies

**Figure 1:**
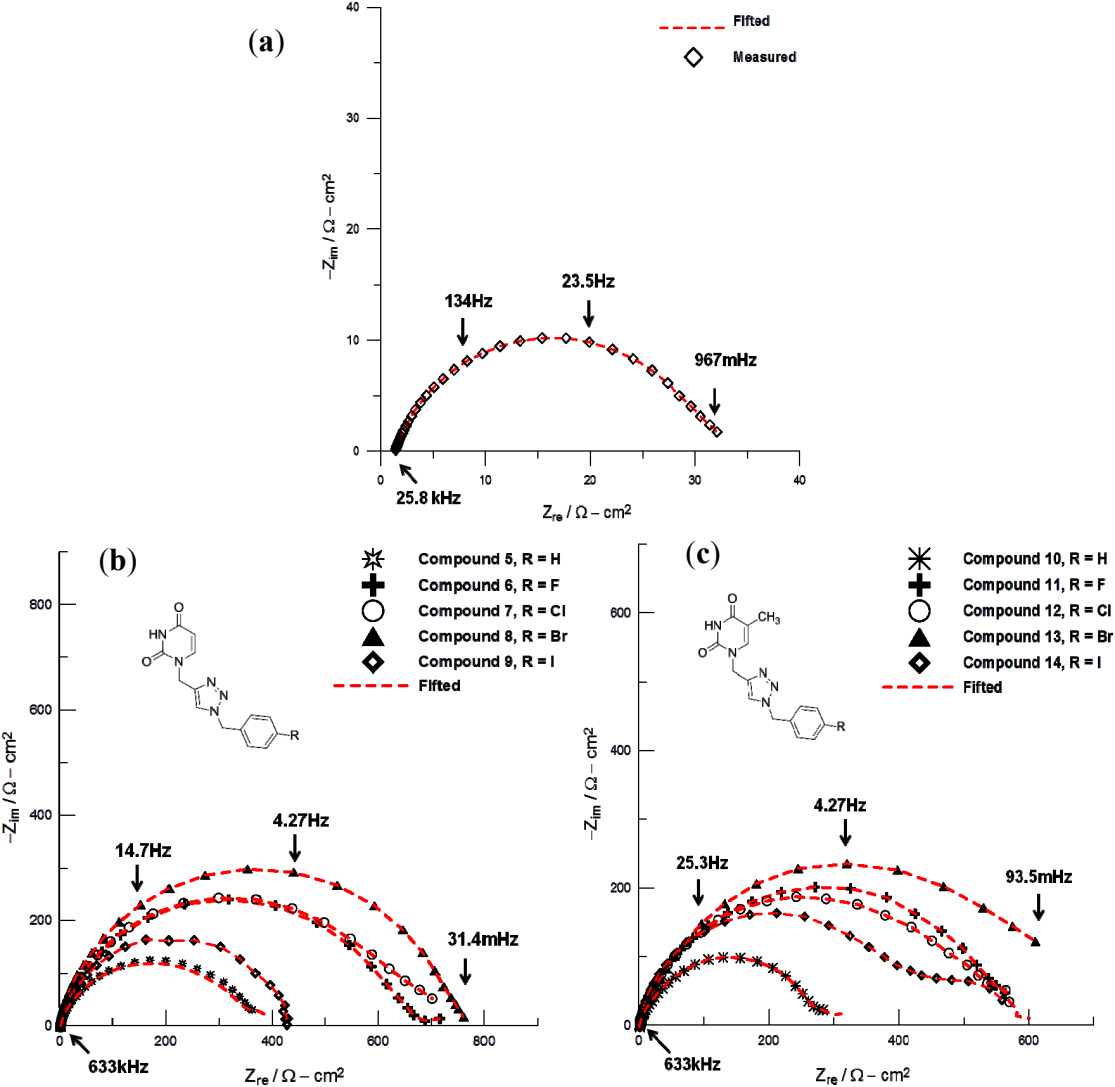
Experimental impedance data, Nyquist plots, recorded in the systems (**a**) API 5L X52/1 M HCl, (**b**) API 5L X52/1 M HCl + 25 ppm of compounds **5-9**, and (**c**) API 5L X52/1 M HCl + 25 ppm of compounds **10**–**14**.

The corrosion inhibitive efficiency, IE, of compounds **5**–**14** was examined by electrochemical impedance spectroscopy. The blank’s response is shown in Figure 1(a). Note that its impedance spectrum exhibited one single depressed semicircle, which indicates that the steel corrosion is mainly controlled by a charge transfer process. In contrast, when compounds **5**–**14** are present (25 ppm), the impedance spectra are characterized, in general, by two time constants (see Figure 1(b) and Figure 1(c)). From these figures it is noted that the spectra obtained after addition of organic molecules to the corroding media increased the impedance (*Z*_re_) value, and that in most of the cases they are characterized by two semicircles or two time constants, one constant at high frequency and the other at low frequency, which are generally attributed to the adsorption of the organic molecules onto the metal surface. The impedance parameters determined from the corresponding Nyquist diagrams are listed in Table 4. Inspection of Table 4 reveals that R_ct_ values increase prominently, while C_dl_ reduces with increasing concentrations of 1,2,3-triazole nucleobases **5**–**14**. A large charge transfer resistance is associated with a slower corroding system. Furthermore, the smaller double layer capacitance, the better protection provided by an inhibitor. It is important to remark that all these compounds displayed corrosion inhibition efficiencies over 90% at rather low concentration values, which resulted even better than other types of corrosion inhibitors reported in the literature [30,32,33,34,35,36,37,38,39]. All compounds studied in this work showed a corrosion inhibitive activity comparable at least or better than to other organic inhibitors derived from purine nucleobases (adenine and guanine) [53,54,55].

**Table 3:** ^1^H, and ^13^C-NMR chemical shifts (ppm) for compounds **10**–**14** in DMSO-*d*_6_. 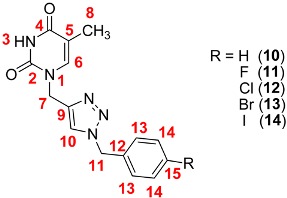

		3-H			6-H	7-H	8-H		10-H	11-H		13-H	14-H	15-H
	2-C		4-C	5-C	6-C	7-C	8-C	9-C	10-C	11-C	12-C	13-C	14-C	15-C
**10**		11.28			7.59	4.85	1.71		8.10	5.54		[a]	[a]	[a]
	151.2		164.8	109.4	141.8	42.8	12.5	143.4	124.2	53.3	136.5	128.5	129.3	128.7
**11**		11.27			7.58	4.85	1.71		8.10	5.53		7.36	7.17	---
	151.2		164.8	109.4	141.7	42.8	12.5	143.4	124.1	52.5	132.7	130.9	116.1	162.4
**12**		11.27			7.59	4.85	1.71		8.10	5.54		7.30	7.40	---
	151.2		164.8	109.4	141.7	42.8	12.5	143.4	124.2	52.5	135.5	130.5	129.3	133.4
**13**		11.30			7.61	4.87	1.73		8.13	5.55		7.26	7.561	---
	151.3		164.8	109.4	141.8	42.8	12.5	143.5	124.3	52.6	135.9	130.8	32.2	122.0
**14**		11.29			7.60	4.87	1.74		8.11	5.53		7.10	7.72	---
	151.3		164.8	109.4	141.7	42.8	12.5	143.4	124.3	52.8	136.2	130.9	138.1	95.1

[a] See Experimental section.

**Table 4:** Electrochemical parameters obtained from experimental impedance data, including the corrosion inhibition efficiencies (IE) at 25 ppm of the organic inhibitor.

Compound	R_s_/Ω cm^2^	R_ct_/Ω cm^2^	C_dl_/μF cm^2^	IE/%
Blank	0.8	30	310	---
5	2.5	435	39	93.1
6	1.3	681	43	95.6
7	1.1	725	50	95.9
8	1.0	770	18	96.1
9	1.7	425	70	92.9
10	1.5	306	19	90.2
11	1.4	600	29	95.0
12	1.4	599	56	95.0
13	1.5	600	54	95.0
14	1.3	588	49	94.9

## 3. Experimental

### 3.1. General

Commercially available reagents and solvents were used as received. Flash column chromatography was performed on Kieselgel silica gel 60 (230–400 mesh). Melting points were determined on a Fisher-Johns apparatus and were uncorrected. IR spectra were recorded on a Bruker Alpha FT-IR/ATR spectrometer (Leipzig, Germany). NMR spectra were obtained with JEOL ECA-500 (500 MHz) and JEOL Eclipse-400 (400 MHz) spectrometers (Tokyo, Japan). Chemical shifts (δ) are given in ppm downfield from Me_4_Si as an internal reference; coupling constants are given in *J* (Hertz). High-resolution mass spectra (HRMS) were recorded on JEOL JMS-SX 102a and Agilent-MSD-TOF-1069A spectrometers (Tokyo, Japan). Compounds **5** and **10** are known, however their spectroscopic data was not reported [56]. The electrochemical impedance study was performed at room temperature using the ZENNIUM-ZAHNER electrochemical workstation (ZAHNER-Electrik GmbH & Co.KG, Kronach, Germany), applying a sinusoidal ± 10 mV perturbation, within the frequency range of 100 KHz to 0.1 Hz to an electrochemical cell with a three-electrode setup. A saturated Ag/AgCl mini-electrode was used as reference, with a graphite bar as counter electrode, while the working electrode was the API 5L X52 steel sample with an exposed area of approximately 1 cm^2^, which was prepared using standard metallographic procedures. The corrosion inhibition efficiency (IE) was evaluated by means of electrochemical impedance spectroscopy (EIS) in the API 5L X52/1 M HCl system containing 0 (blank) or 25 ppm of the organic inhibitor. Simulation of the impedance data recorded was conducted by means of electrical equivalent circuits [40] and the electrical parameters: solution resistance (R_s_), charge transfer resistance (R_ct_), and double layer capacitance (C_dl_) were obtained in this way.

### 3.2. Product Synthesis and Characterization

*1-(Prop-2-ynyl)pyrimidine-2,4(1H,3H)-dione* (**3**). In a 50 mL three-necked round-bottomed flask equipped with a magnetic stirrer, a thermometer, and a reflux condenser, uracil (**1**, 1.12 g, 10 mmol) was suspended in dry acetonitrile (15 mL), *N*,*O*-bis-(trimethylsilyl)acetamide (BSA, 6.12 mL, 25 mmol) was added and the mixture stirred for a few minutes until a clear solution was obtained. Subsequently, propargyl bromide (80 wt.% in toluene, 1.23 mL, 13.8 mmol) was added and the whole reaction mixture was heated at 45 °C for 72 h. The acetonitrile was evaporated under vacuum and the residue was treated with aqueous NH_4_Cl solution (5%, 20 mL) and extracted with CH_2_Cl_2_ in a continuous liquid-liquid extractor for 12 h. The organic phase was dried with anhydrous Na_2_SO_4_ and concentrated under vacuum. The crude product was purified by recrystallization from CH_2_Cl_2_/hexane (1:2 *v*/*v*) to afford 0.96 g (64% yield) of **3** as a white solid, mp 164–166 °C [Lit. [43] mp 169–170 °C]. ^1^H-NMR (DMSO-*d*_6_, 500 MHz): δ = 3.37 (t, *J* = 2.4 Hz, 1H, C≡C-H), 4.47 (d, *J* = 2.5 Hz, 2H, CH_2_), 5.59 (d, *J* = 7.9 Hz, 1H, CH), 7.65 (d, *J* = 7.9 Hz, 1H, NCH), 11.33 (br, 1H, NH). ^13^C-NMR (DMSO-*d*_6_, 125.76 MHz): δ = 37.1 (CH_2_), 76.4 (≡C-H), 79.0 (C≡), 102.2 (CH), 145.0 (NCH), 150.9 (N_2_C=O), 164.1 (NC=O). FT-IR/ATR ν_max_ cm^−1^: 3240 (≡C-H), 3114, 2990, 2907, 2860, 2806, 2117 (C≡C), 1750 (C=O), 1682 (C=O), 1617, 1456, 1408, 1380, 1328, 1239, 1174. HRMS (ESI-TOF) calculated for C_7_H_6_N_2_O_2_ + H^+^: 151.0502; Found: 151.0503.

*5-Methyl-1-(prop-2-ynyl)pyrimidine-2,4-(1H,3H)-dione* (**4**). The procedure described above was followed to obtain compound **4**, employing thymine (**2**, 1.26 g, 10 mmol), BSA (6.12 mL, 25 mmol), and propargyl bromide (80 wt.% in toluene, 1.23 mL, 13.8 mmol). The reaction mixture was heated at 60 °C for 72 h. The crude product was purified by recrystallization from CH_2_Cl_2_/hexane (1:2 *v*/*v*) to afford 1.43 g (87% yield) of **4** as a white solid, mp 155–157 °C [Lit. [45] mp 157–158 °C]. ^1^H-NMR (DMSO-*d*_6_, 500 MHz): δ = 1.72 (d, *J* = 0.9 Hz, 3H, CH_3_), 3.33 (t, *J* = 2.4 Hz, 1H, ≡C-H), 4.42 (d, *J* = 2.5 Hz, 2H, CH_2_), 7.51 (d, *J* = 1.0 Hz, 1H, NCH), 11.35 (br, 1H, NH). ^13^C-NMR (CDCl_3_, 125.76 MHz): δ = 12.4 (CH_3_), 36.9 (CH_2_), 76.1 (≡C-H), 79.2 (C), 110.0 (CCH_3_), 140.7 (NCH), 150.9 (N_2_C=O), 164.7 (NC=O). FT-IR/ATR ν_max_ cm^−1^: 3250 (≡C-H), 3154, 3088, 3012, 2932, 2892, 2831, 2123 (C≡C), 1701, 1649 (C=O), 1516, 1472, 1422, 1353, 1340, 1243, 1218, 1135. HRMS (ESI-TOF) calculated for C_8_H_8_N_2_O_2_ + H^+^: 165.0658; Found: 165.0661.

*1-((1-Benzyl-1H-1,2,3-triazol-4-yl)methyl)pyrimidine-2,4-(1H,3H)-dione* (**5**). In a 50 mL round-bottomed flask equipped with a magnetic stirrer, were added Cu(OAc)_2_∙H_2_O (3.6 mg, 0.02 mmol, 5 mol%), 1,10-phenanthroline monohydrate (4 mg, 0.02 mmol, 5 mol%), and sodium L-ascorbate (59 mg, 0.3 mmol) in EtOH/H_2_O (2:1 *v*/*v*, 3 mL), followed by stirring for five minutes at room temperature. Subsequently, **3** (60 mg, 0.4 mmol), sodium azide (29 mg, 0.44 mmol), and benzyl chloride (51 μL, 0.44 mmol) were added to the reaction mixture which was stirred during 24 h at room temperature. Afterwards, H_2_O (10 mL) was added to reaction mixture to induce precipitation of the product, which was filtered off, washed with H_2_O, then with hexane and dried under vacuum. The crude product was purified by column chromatography (CH_2_Cl_2_/MeOH 90:10 *v*/*v*) and recrystallized from CH_2_Cl_2_/hexane (1:1 *v*/*v*) to afford 95 mg (84% yield) of **5** as a white solid, mp 215–217 °C. ^1^H-NMR (DMSO-*d*_6_, 500 MHz): δ = 4.89 (s, 2H, CH_2_NC=O), 5.53–5.57 (m, 3H, NCH_2_Ph, CH), 7.27–7.36 (m, 5H, ArH), 7.71 (d, *J* = 7.9 Hz, 1H, NCH), 8.10 (s, 1H, ArH, triazole), 11.29 (br, 1H, NH). ^13^C-NMR (DMSO-*d*_6_, 125.76 MHz): δ = 43.0 (CH_2_NC=O), 53.4 (NCH_2_Ph), 101.8 (CH), 124.2 (ArCH, triazole), 128.5 (2×ArCH), 128.7 (ArCH), 129.3 (2×ArCH), 136.4 (C_ipso_), 143.2 (C_ipso_, triazole), 146.1 (NCH), 151.3 (N_2_C=O), 164.3 (NC=O). FT-IR/ATR ν_max_ cm^−1^: 3156, 3106, 3056, 2953, 2871, 2814, 1754, 1721, 1674, 1630, 1454, 1422. HRMS (ESI-TOF) calculated for C_14_H_13_N_5_O_2_ + H^+^: 284.1142; Found: 284.1145.

*1-((1-(4-Fluorobenzyl)-1H-1,2,3-triazol-4-yl)methyl)pyrimidine-2,4(1H,3H)-dione* (**6**). The procedure described above (using the same quantities of Cu(OAc)_2_∙H_2_O, 1,10-phenanthroline monohydrate, sodium L-ascorbate) was followed to obtain compound **6**, employing **3** (60 mg, 0.4 mmol), NaN_3_ (29 mg, 0.44 mmol), and 4-fluorobenzyl chloride (53 μL, 0.44 mmol). The crude product was purified by column chromatography (CH_2_Cl_2_/MeOH 90:10 *v*/*v*) and recrystallized from CH_2_Cl_2_/hexane (1:1 *v*/*v*) to afford 108 mg (90% yield) of the desired product **6** as a white solid, mp 225–227 °C. ^1^H-NMR (DMSO-*d_6_*, 500 MHz): δ = 4.89 (s, 2H, CH_2_NC=O), 5.53 (s, 2H, NCH_2_Ph), 5.55 (d, *J* = 7.9 Hz, 1H, CH), 7.17 (t, *J* = 8.8 Hz, 2H, ArH), 7.36 (dd, *J* = 5.6, 8.5 Hz, 2H, ArH), 7.71 (d, *J* = 7.9 Hz, 1H, NCH), 8.10 (s, 1H, ArH, triazole), 11.28 (br, 1H, NH). ^13^C-NMR (DMSO-*d_6_*, 125.76 MHz): δ = 43.0 (CH_2_NC=O), 52.5 (NCH_2_Ph), 101.8 (CH), 116.1 (d, ^2^*J*_CF_ = 21.4 Hz, 2×ArCH), 124.1 (ArCH, triazole), 130.9 (d, ^3^*J*_CF_ = 8.8 Hz, 2×ArCH), 132.7 (d, ^4^*J*_CF_ = 2.5 Hz, C_ipso_), 143.3 (C_ipso_, triazole), 146.1 (NCH), 151.3 (N_2_C=O), 162.4 (d, *J*_CF_ = 244.0 Hz, F-C_ipso_), 164.2 (NC=O). FT-IR/ATR ν_max_ cm^−1^: 3156, 3106, 3057, 2954, 2871, 2812, 1761, 1721, 1673, 1630, 1606, 1510, 1453. HRMS (ESI-TOF) calculated for C_14_H_12_F_1_N_5_O_2_ + H^+^: 302.1048; Found: 302.1048.

*1-((1-(4-Chlorobenzyl)-1H-1,2,3-triazol-4-yl)methyl)pyrimidine-2,4(1H,3H)-dione* (**7**). The procedure described above (using the same quantities of Cu(OAc)_2_∙H_2_O, 1,10-phenanthroline monohydrate, sodium L-ascorbate) was followed to obtain compound **7**, employing **3** (60 mg, 0.4 mmol), NaN_3_ (29 mg, 0.44 mmol), and 4-chlorobenzyl chloride (74 mg, 0.46 mmol). The crude product was purified by column chromatography (CH_2_Cl_2_/MeOH 90:10 *v*/*v*) and recrystallized from CH_2_Cl_2_/hexane (1:1 *v*/*v*) to afford 102 mg (80% yield) of the desired product **7** as a white solid, mp 222–224 °C. ^1^H-NMR (DMSO-*d_6_*, 500 MHz): δ = 4.89 (s, 2H, CH_2_NC=O), 5.54 (s, 2H, NCH_2_Ph), 5.55 (d, *J* = 7.8 Hz, 1H, CH), 7.30 (d, *J* = 8.7 Hz, 2H, ArH), 7.40 (d, *J* = 8.6 Hz, 2H, ArH), 7.71 (d, *J* = 7.9 Hz, 1H, NCH), 8.11 (s, 1H, ArH, triazole), 11.28 (br, 1H, NH). ^13^C-NMR (DMSO-*d_6_*, 125.76 MHz): δ = 43.0 (CH_2_NC=O), 52.5 (NCH_2_Ph), 101.8 (CH), 124.3 (ArCH, triazole), 129.3 (2×ArCH), 130.5 (2×ArCH), 133.4 (Cl-C_ipso_), 135.4 (C_ipso_), 143.3 (C_ipso_, triazole), 146.1 (NCH), 151.3 (N_2_C=O), 164.2 (NC=O). FT-IR/ATR ν_max_ cm^−1^: 3152, 3100, 3055, 2950, 2869, 2815, 1720, 1675, 1630, 1491, 1452, 831, 788. HRMS (ESI-TOF) calculated for C_14_H_12_Cl_1_N_5_O_2_ + H^+^: 318.0752; Found: 318.0757.

*1-((1-4-Bromobenzyl-1H-1,2,3-triazol-4-yl)methyl)pyrimidine-2,4(1H,3H)-dione* (**8**). The procedure described above (using the same quantities of Cu(OAc)_2_∙H_2_O, 1,10-phenanthroline monohydrate, sodium l-ascorbate) was followed to obtain compound **8**, employing **3** (60 mg, 0.4 mmol), NaN_3_ (29 mg, 0.44 mmol), and 4-bromobenzyl bromide (110 mg, 0.44 mmol). The crude product was purified by column chromatography (CH_2_Cl_2_/MeOH 90:10 *v*/*v*) and recrystallized from CH_2_Cl_2_-Hexane (1:1 *v*/*v*) to afford 120 mg (83% yield) of the desired product **8** as a white solid, mp 241–243 °C. ^1^H-NMR (DMSO-*d_6_*, 500 MHz): δ = 4.88 (s, 2H, CH_2_NC=O), 5.52 (s, 2H, NCH_2_Ph), 5.55 (d, *J* = 7.9 Hz, 1H, CH), 7.23 (d, *J* = 8.2 Hz, 2H, ArH), 7.53 (d, *J* = 8.2 Hz, 2H, ArH), 7.70 (d, *J* = 7.9 Hz, 1H, NCH), 8.11 (s, 1H, ArH, triazole), 11.29 (br, 1H, NH). ^13^C-NMR (DMSO-*d_6_*, 125.76 MHz): δ = 43.0 (CH_2_NC=O), 52.6 (NCH_2_Ph), 101.8 (CH), 122.0 (Br-C_ipso_), 124.3 (ArCH, triazole), 130.8 (2×ArCH), 132.2 (2×ArCH), 135.8 (C_ipso_), 143.3 (C_ipso_, triazole), 146.1 (NCH), 151.3 (N_2_C=O), 164.3 (NC=O). FT-IR/ATR ν_max_ cm^−1^: 3151, 3098, 3054, 2949, 2871, 2815, 1721, 1675, 1630, 1488, 1453, 1422, 831, 787. HRMS (ESI-TOF) calculated for C_14_H_12_Br_1_N_5_O_2_ + H^+^: 362.0247; Found: 362.0247.

*1-((1-(4-Iodobenzyl)-1H-1,2,3-triazol-4-yl)methyl)pyrimidine-2,4-(1H,3H)-dione* (**9**). The procedure described above was followed to obtain compound **9**, employing Cu(OAc)_2_∙H_2_O (3.0 mg, 0.017 mmol), 1,10-phenanthroline monohydrate (3.4 mg, 0.025 mmol), sodium L-ascorbate (52 mg, 0.26 mmol), **3** (50 mg, 0.33 mmol), NaN_3_ (23 mg, 0.36 mmol), and 4-iodobenzyl bromide (113 mg, 0.38 mmol). The crude product was purified by column chromatography (CH_2_Cl_2_/MeOH 90:10 *v*/*v*) and recrystallized from CH_2_Cl_2_/Hexane (1:1 *v*/*v*) to afford 110 mg (81% yield) of the desired product **9** as a white solid, mp 259–261 °C. ^1^H-NMR (DMSO-*d*_6_, 500 MHz): δ = 4.88 (s, 2H, CH_2_NC=O), 5.50 (s, 2H, NCH_2_Ph), 5.55 (d, *J* = 7.9 Hz, 1H, CH), 7.08 (d, *J* = 8.2 Hz, 2H, ArH), 7.70 (d, *J* = 8.2 Hz, 3H, NCH, ArH), 8.10 (s, 1H, ArH, triazole), 11.28 (br, 1H, NH). ^13^C-NMR (DMSO-*d*_6_, 125.76 MHz): δ = 43.0 (CH_2_NC=O), 52.7 (NCH_2_Ph), 95.1 (I-C_ipso_), 101.8 (CH), 124.3 (ArCH, triazole), 130.8 (2×ArCH), 136.2 (C_ipso_), 138.1 (2×ArCH), 143.2 (C_ipso_, triazole), 146.1 (NCH), 151.3 (N_2_C=O), 164.3 (NC=O). FT-IR/ATR ν_max_ cm^−1^: 3100, 3055, 2950, 2868, 2810, 1719, 1675, 1630, 1484, 1453, 1392, 831, 786. HRMS (ESI-TOF) calculated for C_14_H_12_I_1_N_5_O_2_ + H^+^: 410.0108; Found: 410.0108.

*1-((1-Benzyl-1H-1,2,3-triazol-4-yl)methyl)-5-methylpyrimidine-2,4-(1H,3H)-dione* (**10**). The procedure described above was followed to obtain compound **10**, employing Cu(OAc)_2_∙H_2_O (4.0 mg, 0.022 mmol), 1,10-phenanthroline monohydrate (4.4 mg, 0.022 mmol), sodium L-ascorbate (65 mg, 0.33 mmol), **4** (70 mg , 0.43 mmol), NaN_3_ (31 mg, 0.47 mmol), and benzyl chloride (54 μL, 0.47 mmol). The crude product was purified by column chromatography (CH_2_Cl_2_/MeOH 90:10 *v*/*v*) and recrystallized from CH_2_Cl_2_/Hexane (1:1 *v*/*v*) to afford 103 mg (81% yield) of the desired product **10** as a white solid, mp 247–249 °C. ^1^H-NMR (DMSO-*d*_6_, 500 MHz): δ = 1.71 (d, *J* = 1.0 Hz, 3H, CH_3_), 4.85 (s, 2H, CH_2_NC=O), 5.54 (s, 2H, NCH_2_Ph), 7.27–7.35 (m, 5H, ArH), 7.59 (d, *J* = 1.2 Hz, 1H, NCH), 8.10 (s, 1H, ArH, triazole), 11.28 (br, 1H, NH). ^13^C-NMR (DMSO-*d*_6_, 125.76 MHz): δ = 12.5 (CH_3_), 42.8 (CH_2_NC=O), 53.3 (NCH_2_Ph), 109.4 (CCH_3_), 124.2 (ArCH, triazole), 128.5 (2×ArCH), 128.7 (ArCH), 129.3 (2×ArCH), 136.5 (C_ipso_), 141.8 (NCH), 143.4 (C_ipso_, triazole), 151.2 (N_2_C=O), 164.8 (NC=O). FT-IR/ATR ν_max_ cm^−1^: 3121, 3078, 3026, 2836, 1685, 1644, 1441, 730, 705. HRMS (ESI-TOF) calculated for C_15_H_15_N_5_O_2_ + H^+^: 298.1299; Found: 298.1301.

*1-((1-(4-Fluorobenzyl)-1H-1,2,3-triazol-4-yl)methyl)-5-methylpyrimidine-2,4-(1H,3H)-dione* (**11**). The procedure described above was followed to obtain compound **11**, employing Cu(OAc)_2_∙H_2_O (3.0 mg, 0.018 mmol), 1,10-phenanthroline monohydrate (3.6 mg, 0.018 mmol), sodium L-ascorbate (53 mg, 0.27 mmol), **4** (60 mg, 0.37 mmol), NaN_3_ (27 mg, 0.41 mmol), and 4-fluorobenzyl chloride (49 μL, 0.41 mmol). The crude product was purified by column chromatography (CH_2_Cl_2_/MeOH 90:10 *v*/*v*) and recrystallized from CH_2_Cl_2_-Hexane (1:1 *v*/*v*) to afford 104 mg (90% yield) of the desired product **11** as a white solid, mp 249–251 °C. ^1^H-NMR (DMSO-*d*_6_, 500 MHz): δ = 1.71 (d, *J* = 1.2 Hz, 3H, CH_3_), 4.85 (s, 2H, CH_2_NC=O), 5.53 (s, 2H, NCH_2_Ph), 7.17 (t, *J* = 8.9 Hz, 2H, ArH), 7.36 (dd, *J* = 5.4, 8.8 Hz, 2H, ArH), 7.58 (d, *J* = 1.2 Hz, 1H, NCH), 8.10 (s, 1H, ArH, triazole), 11.27 (br, 1H, NH). ^13^C-NMR (DMSO-*d*_6_, 125.76 MHz): δ = 12.5 (CH_3_), 42.8 (CH_2_NC=O), 52.5 (NCH_2_Ph), 109.4 (CCH_3_), 116.1 (d, ^2^*J*_CF_ = 22.6 Hz, 2×ArCH), 124.1 (ArCH, triazole), 130.9 (d, ^3^*J*_CF_ = 8.8 Hz, 2×ArCH), 132.7 (d, ^4^*J*_CF_ = 3.8 Hz, C_ipso_), 141.7 (NCH), 143.4 (C_ipso_, triazole), 151.2 (N_2_C=O), 162.4 (d, *J*_CF_ = 244.0 Hz, F-C_ipso_), 164.8 (NC=O). FT-IR/ATR ν_max_ cm^−1^: 3175, 3110, 3063, 3046, 2811, 1681, 1644, 1603, 1509, 1462, 1214, 780, 758. HRMS (ESI-TOF) calculated for C_15_H_14_F_1_N_5_O_2_ + H^+^: 316.1204; Found: 316.1209.

*1-((1-(4-Chlorobenzyl)-1H-1,2,3-triazol-4-yl)methyl)-5-methylpyrimidine-2,4-(1H,3H)-dione* (**12**). The procedure described above was followed to obtain compound **12**, employing Cu(OAc)_2_∙H_2_O (3.0 mg, 0.018 mmol), 1,10-phenanthroline monohydrate (3.6 mg, 0.018 mmol), sodium L-ascorbate (53 mg, 0.27 mmol), **4** (60 mg, 0.37 mmol), NaN_3_ (27 mg, 0.41 mmol), and 4-chlorobenzyl chloride (69 mg, 0.43 mmol). The crude product was purified by column chromatography (CH_2_Cl_2_/MeOH 90:10 *v*/*v*) and recrystallized from CH_2_Cl_2_-Hexane (1:1 *v*/*v*) to afford 105 mg (87% yield) of the desired product **12** as a white solid, m.p. 245–247 °C. ^1^H-NMR (DMSO-*d*_6_, 500 MHz): δ = 1.71 (s, 3H, CH_3_), 4.85 (s, 2H, CH_2_NC=O), 5.54 (s, 2H, NCH_2_Ph), 7.30 (d, *J* = 8.3 Hz, 2H, ArH), 7.40 (d, *J* = 8.4 Hz, 2H, ArH), 7.59 (s, 1H, NCH), 8.10 (s, 1H, ArH, triazole), 11.27 (br, 1H, NH). ^13^C-NMR (DMSO-*d*_6_, 125.76 MHz): δ = 12.5 (CH_3_), 42.8 (CH_2_NC=O), 52.5 (NCH_2_Ph), 109.4 (CCH_3_), 124.2 (ArCH, triazole), 129.3 (2×ArCH), 130.5 (2×ArCH), 133.4 (Cl-C_ipso_), 135.5 (C_ipso_), 141.7 (NCH), 143.4 (C_ipso_, triazole), 151.2 (N_2_C=O), 164.8 (NC=O). FT-IR/ATR ν_max_ cm^−1^: 3124, 3081, 3032, 2833, 1680, 1645, 1491, 1462, 1212, 779, 762. HRMS (ESI-TOF) calculated for C_15_H_14_Cl_1_N_5_O_2_ + H^+^: 332.0909; Found: 332.0911.

*1-((1-(4-Bromobenzyl)-1H-1,2,3-triazol-4-yl)methyl)-5-methylpyrimidine-2,4-(1H,3H)-dione* (**13**). The procedure described above was followed to obtain compound **13**, employing Cu(OAc)_2_∙H_2_O (3.0 mg, 0.018 mmol), 1,10-phenanthroline monohydrate (3.6 mg, 0.018 mmol), sodium L-ascorbate (53 mg, 0.27 mmol), **4** (60 mg, 0.37 mmol), NaN_3_ (27 mg, 0.41 mmol), and 4-bromobenzyl bromide (102 mg, 0.41 mmol). The crude product was purified by column chromatography (CH_2_Cl_2_/MeOH 90:10 *v*/*v*) and recrystallized from CH_2_Cl_2_-Hexane (1:1 *v*/*v*) to afford 115 mg (83% yield) of the desired product **13** as a white solid, mp 245–247 °C. ^1^H-NMR (DMSO-*d*_6_, 400 MHz): δ = 1.73 (s, 3H, CH_3_), 4.87 (s, 2H, CH_2_NC=O), 5.55 (s, 2H, NCH_2_Ph), 7.26 (d, *J* = 7.7 Hz, 2H, ArH), 7.56 (d, *J* = 7.7 Hz, 2H, ArH), 7.61 (s, 1H, NCH), 8.13 (s, 1H, ArH, triazole), 11.30 (br, 1H, NH). ^13^C-NMR (DMSO-*d*_6_, 100.5 MHz): δ = 12.5 (CH_3_), 42.8 (CH_2_NC=O), 52.6 (NCH_2_Ph), 109.4 (CCH_3_), 122.0 (Br-C_ipso_), 124.3 (ArCH, triazole), 130.8 (2×ArCH), 132.2 (2×ArCH), 135.9 (C_ipso_), 141.8 (NCH), 143.5 (C_ipso_, triazole), 151.3 (N_2_C=O), 164.8 (NC=O). FT-IR/ATR ν_max_ cm^−1^: 3123, 3080, 3034, 2835, 1684, 1646, 1465, 1214, 762. HRMS (ESI-TOF) calculated for C_15_H_14_Br_1_N_5_O_2_ + H^+^: 376.0404; Found: 376.0407.

*1-((1-(4-Iodobenzyl)-1H-1,2,3-triazol-4-yl)methyl)-5-methylpyrimidine-2,4-(1H,3H)-dione* (**14**). The procedure described above was followed to obtain compound **14**, employing Cu(OAc)_2_∙H_2_O (2.7 mg, 0.015 mmol), 1,10-phenanthroline monohydrate (3.0 mg, 0.015 mmol), sodium L-ascorbate (46 mg, 0.27 mmol), **4** (50 mg, 0.30 mmol), NaN_3_ (21 mg, 0.33 mmol), and 4-iodobenzyl bromide (104 mg, 0.35 mmol). The crude product was purified by column chromatography (CH_2_Cl_2_/MeOH 90:10 *v*/*v*) and recrystallized from CH_2_Cl_2_/Hexane (1:1 *v*/*v*) to afford 110 mg (85% yield) of the desired product **14** as a white solid, mp 238–240 °C. ^1^H-NMR (DMSO-*d*_6_, 400 MHz): δ = 1.74 (s, 3H, CH_3_), 4.87 (s, 2H, CH_2_NC=O), 5.53 (s, 2H, NCH_2_Ph), 7.10 (d, *J* = 7.9 Hz, 2H, ArH), 7.60 (s, 1H, NCH), 7.72 (d, *J* = 8.2 Hz, 2H, ArH), 8.11 (s, 1H, ArH, triazole), 11.29 (br, 1H, NH). ^13^C-NMR (DMSO-*d*_6_, 100.5 MHz): δ = 12.5 (CH_3_), 42.8 (CH_2_NC=O), 52.8 (NCH_2_Ph), 95.1 (I-C_ipso_), 109.4 (CCH_3_), 124.3 (ArCH, triazole), 130.9 (2×ArCH), 136.2 (C_ipso_), 138.1 (2×ArCH), 141.7 (NCH), 143.4 (C_ipso_, triazole), 151.3 (N_2_C=O), 164.8 (NC=O). FT-IR/ATR ν_max_ cm^−1^: 3161, 3136, 3088, 3040, 2821, 1684, 1648, 1464, 1216, 777, 759. HRMS (ESI-TOF) calculated for C_15_H_14_I_1_N_5_O_2_ + H^+^: 424.0265; Found: 424.0264.

## 4. Conclusions

The synthetic protocol for the preparation of *N*-1-propargylpyrimidine nucleobases has been optimized, these derivatives are important building blocks for the synthesis of many 1,2,3-triazoles of interest. Eight new 1,2,3-triazole derivatives of pyrimidine nucleobases were successfully synthesized in good yields through a one-pot three-component click reaction and fully characterized. The electrochemical study evidenced that these new class of heterocyclic compounds are promising corrosion inhibitors of steel in 1 M hydrochloric acid.
